# Deleting *Bmp4* in brown fat unlocks an arachidonic acid-AMPK axis to boost energy expenditure

**DOI:** 10.1016/j.jlr.2026.101103

**Published:** 2026-07-15

**Authors:** Yan-Jue Song, Ting Meng, Zhen-Yu Xu, Ting Zhang, Zhao-Ning Wang, Dai-Chen Yao, Li-Jie Yang, Ping Wu, Shu-Wen Qian, Yang Liu, Qi-Qun Tang, Yan Tang

**Affiliations:** 1 Key Laboratory of Metabolism and Molecular Medicine, Ministry of Education, Department of Biochemistry and Molecular Biology of School of Basic Medical Sciences and Department of Endocrinology and Metabolism of Zhongshan Hospital, Fudan University, Shanghai, China; 2 Department of Laboratory Medicine, Shanghai Fourth Rehabilitation Hospital, Shanghai, China

**Keywords:** Adipose tissue, Lipids/Oxidation, Lipidomics, Mitochondria, Triglycerides, Re-esterification, Fasting, Fatty acid metabolism

## Abstract

Fasting enhances lipolysis in white adipose tissue (WAT), releasing fatty acids (FAs) which are subsequently metabolized through β-oxidation in the liver and brown adipose tissue (BAT). Although BAT is a key site for FA utilization, the specific FAs taken up and oxidized during fasting-as well as their systemic metabolic effects-remain poorly defined. Time-course analysis revealed that fasting reprograms BAT to promote the esterification and storage of circulating FAs rather than their immediate oxidation. This lipid pool was selectively enriched in long-chain polyunsaturated fatty acids (LCPUFAs), particularly arachidonic acid (AA). Genetic (BAT-specific knockout of bone morphogenetic protein 4, *Bmp4*) or pharmacological (Inhibition of diacylglycerol acyltransferase, DGAT) disruption of this FA esterification process potently enhanced mitochondrial function and systemic energy expenditure. Mechanistically, AA activated AMP-activated protein kinase (AMPK), thereby enhancing energy expenditure through increased mitochondrial biogenesis. Our work reveals a fasting-inducible, AA-centric signaling pathway that senses lipid storage status. Through activation of AMPK, it couples suppressed FA esterification to enhanced oxidation, positioning BMP4 as a critical regulator of this metabolic switch.

## Graphical abstract

**Figure:**
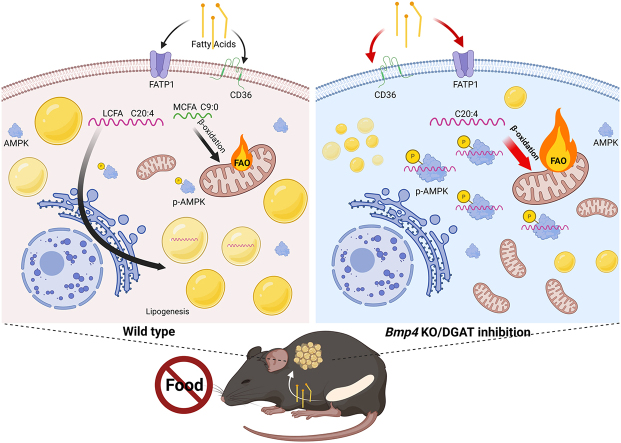

Fasting has emerged as a promising intervention to alleviate obesity-associated metabolic disorders and to extend lifespan in various model organisms (1, 2, 3). Systemic benefits include enhanced white adipose tissue (WAT) browning (4, 5, 6), improved hepatic metabolism in metabolic dysfunction-associated steatohepatitis (MASH) (7, 8), and cardiometabolic improvements (9, 10).

Critically, many of these benefits are mediated through inter-organ communication, particularly the metabolic crosstalk between WAT and brown adipose tissue (BAT). During fasting, WAT undergoes lipolysis, releasing FAs that serve as crucial energy substrates for peripheral tissues, with BAT being a key consumer (11, 12). In response, BAT shifts its energy substrate preference from glucose to FAs (13). This uptake presents BAT with a fundamental metabolic decision: internalized FAs can be esterified into triglycerides (TG) for storage within lipid droplets (14, 15) or directed into mitochondria for β-oxidation to fuel thermogenesis (16, 17, 18). How BAT allocates FAs between these divergent fates under fasting conditions is a central, unresolved question.

While it was traditionally held that FAs utilized by BAT originated primarily from WAT (19, 20), accumulating evidence now underscores the essential contribution of intracellular lipid stores within BAT itself to thermogenesis, as ablation of key lipases (e.g., adipose TG lipase and hormone-sensitive lipase) impairs thermogenic function (21). However, we observed that during a 20-h fast, BAT markedly increased lipid droplet accumulation, which is an adaptation reminiscent of physiological hepatic steatosis (22). This finding raises a key mechanistic question: What is the role of lipid synthesis in subsequent lipid breakdown, and how does it regulate thermogenesis? Intriguingly, emerging evidence suggests that inhibiting lipid formation may promote lipolysis and support the maintenance of body temperature (23, 24). Uncoupling protein 1 (*Ucp1*)-driven diacylglycerol O-acyltransferase (*Dgat*)-knockout mice maintain normal body temperature at 4°C (19). Furthermore, mice with BAT-specific AKT kinase 2 (*Akt2*) deletion show impaired lipid accumulation yet mildly elevated body temperature after cold exposure (23). Thus, it is necessary to revisit the physiological role of BAT lipid storage in a different physiological or metabolic stress model.

Bone morphogenetic protein 4 (BMP4) is a well-established regulator of adipose tissue development and plasticity. In addition to regulating mesenchymal stem cell (MSC) commitment toward adipogenic lineage and differentiation into mature adipocytes (25), BMP4 induces WAT browning and decreases lipid accumulation (26). Conversely, BMP4 has also been implicated in promoting the "whitening" of BAT, a process associated with increased lipid storage capacity (26, 27). Thus, the paradox that *Bmp4* overexpression causes opposite effects in WAT and BAT lacks a unified explanation, hinting at a more complex regulatory logic. The previous study has shown that *Bmp4* overexpression enhances lipid droplet size without inhibiting β_3_-adrenergic receptor-stimulated lipolysis, pointing to a primary effect on lipid synthesis (27). In WAT, BMP4 upregulates the lipogenic gene stearoyl-CoA desaturase 1 (*Scd1*) (28). However, it is not clear if BMP4 directly stimulates de novo lipogenesis in BAT, nor whether it is involved in the physiological expansion of lipid stores during fasting. Based on this gap, we hypothesized that BMP4 acts as a key arbitrator of fatty acid fate decisions in BAT under fasting conditions.

In this study, we found that deletion of *Bmp4* in BAT (*Bmp4*^Δ*Ucp1*^ mice) inhibited fasting-induced lipid droplet formation but surprisingly enhanced FA oxidation. Mechanistically, arachidonic acid (AA) acts as a key lipid mediator. Under physiological conditions, BMP4 promotes AA esterification into TG. When FA re-esterification is blocked, liberated AA activates AMPK signaling, thereby redirecting metabolic flux toward mitochondrial β-oxidation. Our findings elucidate a BMP4-AA-AMPK axis that dynamically regulates the fate of FAs in BAT, determining whether they are stored for future use or immediately oxidized for energy. Thus, we identified BMP4 as a fasting-responsive regulator that mediates lipid metabolism in BAT during fasting.

## Materials and methods

### Animal experiments design

All animal experiments were performed in accordance with the National Institutes of Health *Guide for the Care and Use of Laboratory Animals* and were approved by the Animal Ethics Committee of Shanghai Medical College, Fudan University. *Bmp4*-BAT specific knock out mice (*Bmp4*^Δ*Ucp1*^) were generated as previously described (29). The mice were bred in a specific pathogen-free facility in the Laboratory Animal Center of Fudan University and housed on a 12-h light:12-h dark cycle at 22–24°C with 40%–70% humidity and ad libitum access to food and water.

For fasting and refeeding scheme, mice were subjected to a 12-h fast starting at 7:00 a.m., followed by a 2-h intensive refeeding period to maximize whole-body glycogen reserves, as previously reported (30). Mice were randomly assigned to different fasting time points or a refeeding 4 h group. Only the same gender and similar age littermate mice were used as the control group.

### Cell culture and adipogenic induction

Immortalized brown preadipocytes were generated and validated as previously described (31). Cells were cultured in DMEM (Meilunbio Inc, Cat#MA0212-2) supplemented with 10% heat-inactivated FBS (Sino Biological, Cat#FBS05) and 1% penicillin-streptomycin. All cell lines were maintained at 37°C with 5% CO2 in a humidified incubator. Cells were plated at 70% confluency and initially treated for 2 days with differentiation medium (DMEM containing 10% FBS, 10 μg/ml insulin (Yeasen Biotechnology, Cat#40112ES60), and 2 nM triiodothyronine). Subsequently, cells were induced for 2 days using induction medium (differentiation medium plus 1 μM dexamethasone (Sigma, Cat#D4902-25MG), 0.5 mM IBMX (Sigma, Cat#410957-1GM), and 0.125 mM indomethacin), followed by 4 days of alternating differentiation medium treatments every other day to achieve full differentiation.

### Bulk RNA-seq library preparation and analysis

Total RNA was purified using TRIzol reagent (Invitrogen, Cat#15596026CN) from BAT of *Bmp4*^fl/fl^ and *Bmp4*^ΔUcp1^ mice. RNA quality was assessed (NanoDrop ND-1000, Agilent 2100 Bioanalyzer with RIN >7.0). Poly(A) RNA was enriched from 1 μg total RNA with two rounds of Dynabeads Oligo(dT) purification (Thermo Fisher, 25-61005). Following fragmentation (NEB Magnesium RNA Fragmentation Module, 94°C), RNA was reverse transcribed with SuperScript II (Invitrogen, Cat#1896649). Second-strand synthesis was performed with E. coli DNA polymerase I, RNase H (NEB), and dUTP solution (Thermo Fisher, R0133) for U-labeling. After PCR amplification, library (average insert 300 ± 50 bp) was sequenced on an Illumina NovaSeq 6000 platform (Novogene Technology) in PE150 mode.

Raw reads were processed with FastQC and Cutadapt (min length 30 bp), then aligned to the GRCm38.102 genome using STAR. Differential expression analysis (Limma) was followed by GSEA (clusterProfiler, default parameters). Visualizations were created with ggplot2. The workflow combined Linux-based sequence processing and R-based statistical analysis.

### Metabolomics

Polar metabolites were extracted from tissues (BAT, 20 mg) using 500 μl of ice-cold methanol: H_2_O (4:1, v/v) containing 0.224 mM phenylhydrazine. After homogenization, samples were incubated at 1,500 rpm for 30 min at 4°C and then kept at −20°C for 1 h to derivatize α-keto acids. Following derivatization (10 min, 12,000 rpm, 4°C), the supernatant was collected, and the extraction was repeated once. The combined extracts were dried in a SpeedVac (H_2_O mode) and reconstituted in 5% acetonitrile for LC-MS analysis.

Analysis was carried out on an Agilent 1290 II UPLC system coupled to a Sciex 5600+ quadrupole-TOF MS. Separation was performed on a Waters ACQUITY HSS-T3 column (3.0 × 100 mm, 1.8 μm) under reverse-phase conditions. MS detection used an ESI source with voltages of +5.5 kV (positive) and −4.5 kV (negative), vaporizer temperature 500°C, and nitrogen gas pressures of 50 psi (drying/nebulizer) and 35 psi (curtain). The scan range was *m/z* 60–700. MS/MS data were acquired in information-dependent acquisition mode with collision energy of (±) 35 ± 15 eV. Data were collected and processed using Analyst® TF 1.7.1 Software (AB Sciex).

### Lipidomics

For lipid extraction from tissue, samples (30 mg) were extracted according to methyl-tert-butyl ether (MTBE) method. Lipidomics analysis was performed by LC-MS/MS using reverse-phase chromatography on a CSH C18 column (1.7 μm, 2.1 × 100 mm; Waters) at 45°C with a flow rate of 300 μl/min. MS detection was carried out on a Q Exactive mass spectrometer (Thermo Fisher Scientific) with electrospray ionization in positive and negative modes. Lipidsearch (Thermo Fisher Scientific) was used to extract and identify the peaks of lipid molecules and internal standard lipid molecules. The main parameters were as follows: precursor tolerance: 5 ppm, product tolerance: 5 ppm, product ion threshold: 5%. The processed data were analyzed by R package (ropls), where it was subjected to multivariate data analysis.

### RT-qPCR

Total RNA was extracted by VeZol (Vazyme, Cat#R411) and stored in diethyl pyrocarbonate (DEPC) H_2_O at −20°C. Reverse transcription to complementary DNA was performed by HiScript IV RT SuperMix (Vazyme, Cat#R423), according to the manufacturer’s instructions. Quantitative PCR was assessed by ChamQ Universal SYBR qPCR Master Mix (Vazyme, Cat#Q711) with ViiATM 7 Real-Time PCR System (Applied Biosystems). All samples were normalized to 18S. The primers used are shown in the Supplemental Table S1.

### Western blot

Primary antibodies against the following proteins were used: β-tubulin (ProteinTech Group, Cat#66240-1-Ig, 1:10,000), HSL (Cell Signaling Technology, Cat#4107, 1:1000), p-HSL (Cell Signaling Technology, Cat#45804, 1:1000), BMP4 (Millipore, Cat#MAB1049, 1:1000), CD36(Sino Biological, Cat#80263-T48, 1:1000), p-AMPKalpha (Cell Signaling Technology, Cat#2535, 1:1000), AMPKalpha (Cell Signaling Technology, Cat#5832, 1:1000) and GYS1 (Abcam, Cat#ab40810, 1:1000).

## Measurement of oxygen consumption rate

### Seahorse XFe24 analysis

The oxygen consumption rate (OCR) of BAT and brown adipocytes was measured using the Seahorse XFe24 Extracellular Flux Analyzer (Agilent). For tissue, BAT tissues isolated from mice were cut into small pieces and placed into XF24 Islet Capture Microplates (Agilent, Cat#101122-100). For cells, immortalized brown preadipocytes were differentiated in XF24 Cell Culture Microplate (Agilent, Cat#100882-004). Tissues or cells were equilibrated in substrate-limited DMEM (pH 7.4) at 37 °C in a non-CO_2_ incubator and subjected to the mitochondrial stress test by adding oligomycin (10 μM for tissue and 1 μM for cells) followed by carbonyl cyanide4-(trifluoromethoxy) phenylhydrazone (FCCP, 9 μM for tissue and 1 μM for cells) and antimycin/rotenone (7 μM for tissue and 1 μM for cells).

### Clark-type oxygen electrode assay

Cellular oxygen consumption using FA substrates was additionally measured using an Oxygraph + system (Hansatech Instruments) with a Clark-type electrode. Briefly, differentiated brown adipocytes were suspended in 1 ml PBS containing 400 μM palmitic acid (PA) or arachidonic acid (AA) complexed with 2% fatty acid-free BSA, and supplemented with 0.5 mM L-carnitine. The OCR was normalized to total cellular protein quantified by BCA assay.

### Molecular docking simulation

The X-ray crystal structure of human AMPK (PDB ID:4RER) was selected from the PDB database. The 3D structure of AA was downloaded from the PubChem database (CID: 444899). Molecular docking was performed using AutoDock. Water molecules and ligands were removed, and hydrogen atoms were added. The Grid Box was set to encompass the entire AMPK structure. The Genetic Algorithm parameters were configured as follows: Number of docking runs = 100; Maximum number of evaluations (evals) = 10,000,000 (medium); Maximum number of generations = 100,000. The top two results were selected based on a comprehensive analysis of van der Waals forces, desolvation energy (hydrophobic interactions), and the lowest binding energy.

### Animal metabolic measurements

O_2_ consumption, CO_2_ production, and heat production of mice were recorded by Comprehensive Lab Animal Monitoring System (CLAMS; Columbus Instruments). Each mouse was subjected to body composition measurements by NMR analyzer (MiniSpec Live Mice Analyzer; Bruker) and acclimatized in the recording chambers for 24–48 h before experiments. During experiments, mice were provided ad libitum access to food and water during light and dark cycles for 48 h, under room temperature or 4°C cold conditions.

### Glucose tolerance test (GTT) and insulin tolerance test (ITT)

Blood glucose levels were measured using a handheld glucometer (Johnson & Johnson). For the GTT, mice injected intraperitoneally with D-glucose (2 mg/g body weight) after 16 h fasting (5 p.m.-9 a.m.). For the ITT, mice received intraperitoneal injections of insulin (0.75 IU/kg body weight) after 4 h fasting (9 a.m.-1 p.m.). Tail vein blood samples were collected at 0,15,30,60,90 and 120 min post-injection for glucose measurement.

### Blood metabolite measurements

Murine blood samples were collected and centrifuged at 3,000g for 10 min at 4°C to isolate serum following specified fasting/refeeding regimens. Blood metabolites including glucose, TG, TC and FA were analyzed using the Cobas e411 automatic biochemical analysis device (Roche Diagnostic), as previously described (32).

### FA treatment

Differentiated brown adipocytes were serum-starved for 20 h prior to FA treatment. 200 μM or specified concentration of PA (Sigma, Cat#P0500) or AA (MCE, Cat#HY-N0830) was conjugated with 5% FA-free BSA, as adapted from a previous study (33). Cells were treated for the indicated durations and harvested for subsequent analyses.

### Quantification and statistical analysis

All data are shown as the mean ± SD. All statistical analyses were performed using GraphPad Prism 10.1.2 (GraphPad Software). Prior to parametric tests, normality (e.g., Shapiro-Wilk test) and homogeneity of variances (e.g., Brown-Forsythe test or Levene test) were verified to ensure the data met the assumptions of the statistical methods. Statistical significance was evaluated with the two-tailed unpaired Student’s *t* test for comparing two groups and one-way ANOVA for comparing more than two groups; if significant, post hoc tests were conducted using Tukey’s test for pairwise comparisons or Dunnett’s test for comparisons with a control group. For analyses involving two independent variables on one dependent variable, two-way ANOVA was applied, followed by Sidak's or Tukey's multiple comparisons tests for post hoc analysis. Statistical significance was denoted as ∗*P* < 0.05, ∗∗*P* < 0.01, ∗∗∗*P* < 0.001, and ∗∗∗∗*P* < 0.0001, with ns indicating no significant difference.

## Results

### Fasting diverts FA to be re-esterified in BAT rather than WAT

**Fig. 1:**
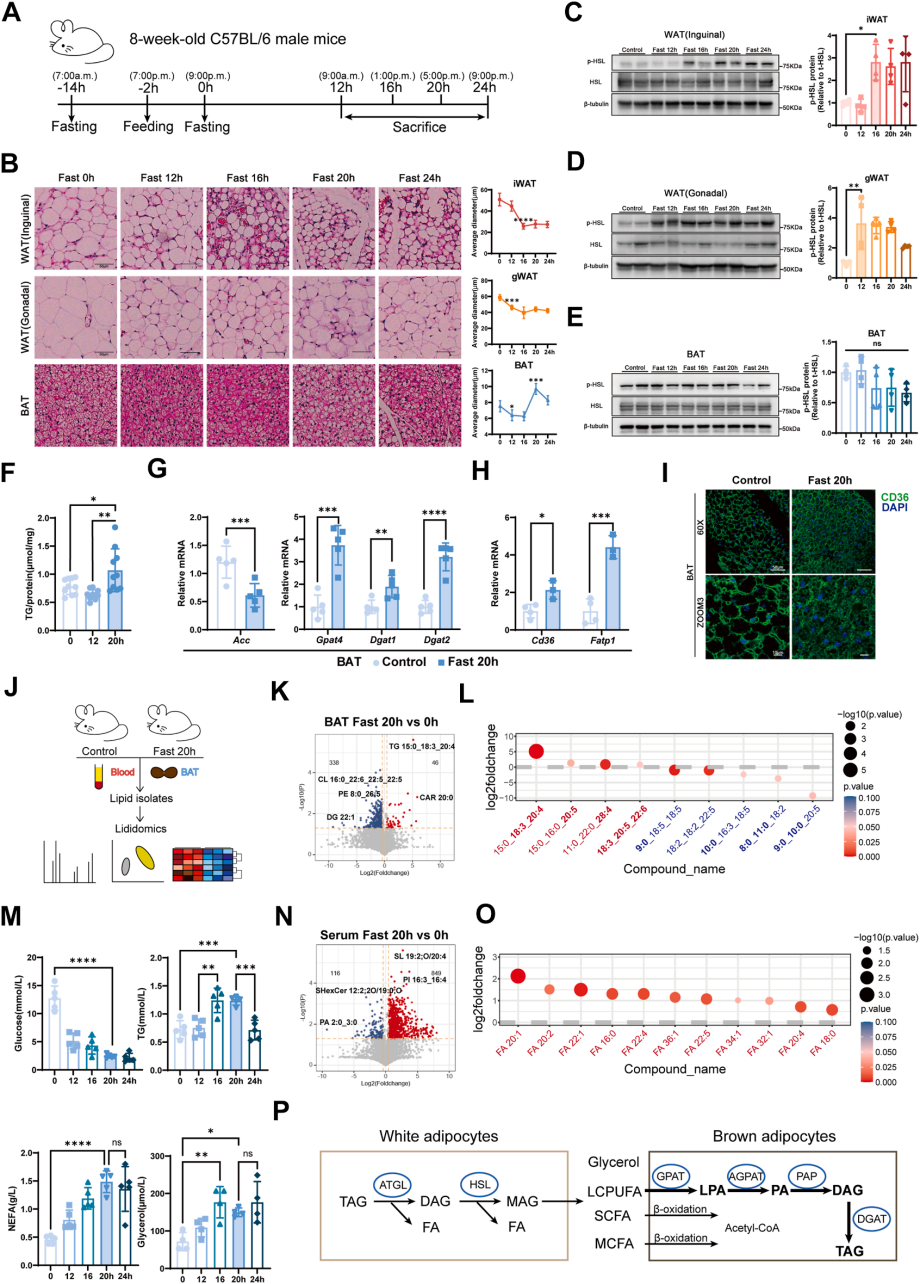
Fasting induces lipolysis of WAT and FA re-esterification of BAT. A: Schematic diagram of the experimental fasting model. B: Representative H&E-staining microscopic images of iWAT, gWAT, and BAT and statistical analysis of lipid droplet diameters (n = 5). C-E: p-HSL and HSL protein expression in iWAT, gWAT and BAT (n = 4). F: Quantification of TG content in BAT (n = 10). G-H: Comparison of mRNA levels for lipogenesis genes (n = 5) and FA uptake genes in BAT (n = 3–4). I: Representative IF image of CD36 in BAT. J: Schematic diagram of the lipidomics protocol. K: Volcano plot of lipidomic profile in BAT from fasted mice and the control (n = 3). L: Relative abundance of TGs in BAT, selected from the lipidomic analysis shown in (K) (n = 3). M: Plasma levels of glucose, TG, NEFA and glycerol in mice (n = 4–5). N: Volcano plot of lipidomic profile in serum comparing fasted mice and the control (n = 3). O: Relative abundance of FAs in serum, selected from the lipidomic analysis shown in (N) (n = 3). P: FA communication and trafficking in WAT and BAT of wild-type mice under fasting conditions. Data are shown as mean ± SD. ∗*P* < 0.05, ∗∗*P* < 0.01, ∗∗∗*P* < 0.001, ∗∗∗∗*P* < 0.0001 by one-way ANOVA (B-E, F and M) and Student’s *t* test (G-H). iWAT, inguinal white adipose tissue; gWAT, gonadal white adipose tissue; BAT, brown adipose tissue; TG, triglyceride; FA, fatty acid; IF, immunofluorescence; NEFA, non-esterified fatty acid.

To accurately assess physiological status after fasting, mice were subjected to a 12-h fast/refeed protocol to standardize their initial metabolic state and followed by a 24-h fasting period (Fig. 1A) according to previous studies (30). As expected, fasting activated lipolysis in both inguinal (iWAT) and gonadal white adipose tissue (gWAT), as evidenced by a reduction in lipid droplet size (Fig. 1B) and elevated levels of phosphorylated hormone-sensitive lipase (p-HSL) (Fig. 1C, D, Supplemental Fig. S1A, B). Notably, p-HSL activation occurred earlier in gWAT (12 h) than in iWAT (16 h), suggesting a depot-specific hierarchy in the mobilization of energy reserves during fasting (34, 35).

In contrast to WAT, BAT displayed a different response. Following fasting, BAT displayed lipid droplet accumulation analogous to physiological hepatic steatosis (Supplemental Fig. S1C–E) (22). More detailed time-course analysis showed that BAT lipid droplet size decreased sharply during the initial 16 h of fasting but markedly increased by 20 h (Fig. 1B, F). Critically, this late-phase lipid accumulation occurred despite stable p-HSL levels (Fig. 1E), indicating that lipolysis was not the main driver of lipid dynamics in BAT. Instead, it suggests that processes of lipid uptake or synthesis predominate. Indeed, qPCR analysis suggested a shift toward FA re-esterification rather than de novo lipogenesis, as key FA re-esterification gene-glycerol-3-phosphate acyltransferase 4 (*Gpat4*), diacylglycerol O-acyltransferase 1 *(Dgat1),* and diacylglycerol O-acyltransferase 2 *(Dgat2)*-were significantly upregulated, whereas the expression of a major de novo lipogenesis gene-acetyl-CoA carboxylase (*Acc*), remained unchanged in BAT after fasting 20 h (Fig. 1G). However, *Ucp1* mRNA expression in BAT did not change after fasting, indicating that this dynamic change in lipid droplet size does not involve UCP1-dependent thermogenesis (Supplemental Fig. S1F). Concurrently, the coordinated induction of FA transporters-fatty acid transport protein 1 (*Fatp1*) and *Cd36* (also known as fatty acid translocase) and CD36 membrane trafficking indicates a programmed enhancement of FA uptake in fasting BAT (36, 37, 38) (Fig. 1H, I).

This fasting-induced lipid accumulation in BAT contrasted sharply with its canonical response to cold (39). Unlike the accumulation observed here, cold exposure alone did not significantly alter BAT lipid droplet size (Supplemental Fig. S1G–I). This comparison clearly indicates that under pure cold stress, the rates of lipolysis and thermogenic consumption outweigh lipid storage, fundamentally differing from the fuel-conserving metabolic reprogramming triggered by energy deprivation alone.

### Fasting remodels the triglyceride landscape of BAT toward long-chain polyunsaturated species

To examine changes in the triglyceride (TG) composition of BAT after 20 h of fasting, we performed untargeted lipidomics on BAT and serum (Fig. 1J). This analysis revealed a profound remodeling of the BAT TG pool. In BAT, levels of 46 TG species increased while 338 decreased after 20 h compared with no fasting (Fig. 1K). Notably, the elevated TG species in BAT were enriched in long-chain polyunsaturated fatty acids (LCPUFA; mostly > 20 carbons), whereas the reduced TG tended to contain medium-chain fatty acids (<12 carbons) (Fig. 1L). Serum analysis mirrored this shift: total TG, FA, and glycerol levels peaked at 20 h (Fig. 1M), and a predominant increase in LCPUFA was also observed (Fig. 1O). The concordance between tissue and circulation indicates that fasting drives a selective uptake and esterification of circulating LCPUFA into the BAT TG pool.

Collectively, these data demonstrate that fasting does not induce a generic lipid accumulation in BAT. Instead, it promotes a specific reprogramming of BAT lipid metabolism characterized by the active re-esterification and storage of externally derived LCPUFA. Alternatively, this selective uptake mechanism may represent an adaptive strategy that enables BAT to maintain its function during nutrient deprivation (Fig. 1P).

### *Bmp4* deletion in BAT prevents lipid accumulation

**Fig. 2:**
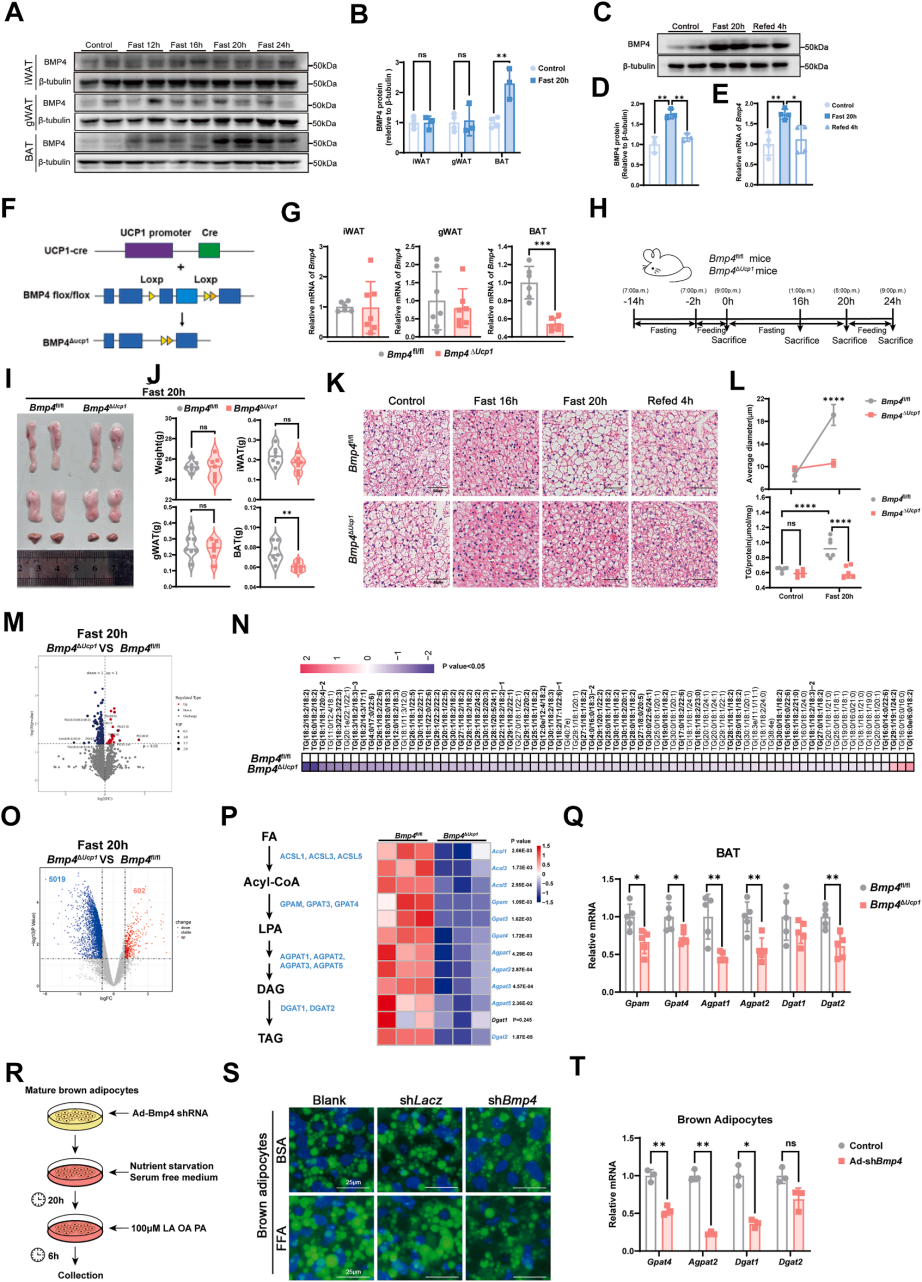
*Bmp4* deletion in BAT prevents lipid accumulation. A-B: BMP4 expression in iWAT, gWAT and BAT from mice fasted for 0, 12, 16, 20, and 24 h (n = 3–4). C-D: BMP4 expression in BAT from mice fasted for 20 h or followed by 4-h refeeding (n = 3). E: Relative mRNA levels of *Bmp4* in BAT (n = 4). F: Schematic of the BAT-specific *Bmp4* knockout (*Bmp4*^Δ*Ucp1*^) mouse model. G: Relative mRNA levels of *Bmp4* in iWAT, gWAT and BAT from *Bmp4*^fl/fl^ mice and *Bmp4*^Δ*Ucp1*^ mice (n = 6–7). H: Schematic diagram of fasting and refeeding model in *Bmp4*^fl/fl^ mice and *Bmp4*^Δ*Ucp1*^ mice. I: Representative images of adipose tissues from mice fasted for 20 h. J: Body weight and weights of fat pads of mice fasted for 20 h (n = 7). K: Representative H&E-staining microscopic images of BAT. L: Quantitative analyses of adipocyte diameter (n = 4) and triglyceride content (n = 6) in BAT. M: Volcano plot of lipidomic profile in BAT from mice fasted for 20 h (n = 5). (N) Relative abundance of TG in BAT, selected from the lipidomic analysis shown in (M) (n = 5). O: Volcano plot of total RNA sequencing from BAT of *Bmp4*^Δ*Ucp1*^ mice versus *Bmp4*^fl/fl^ mice fasted for 20 h (n = 3). P: Heat map of FA esterification genes in BAT (n = 3). Q: Relative mRNA levels of FA esterification genes in BAT (n = 5). R: Schematic of an in vitro cell model exposed to high FA levels to simulate the fasting state. S: Representative BODIPY staining of mature brown adipocytes infected with Ad-sh*LacZ* or Ad-sh*Bmp4* and treated with BSA or FA. T: Relative mRNA levels of FA esterification genes in mature brown adipocytes infected with Ad-sh*LacZ* or Ad-sh*Bmp4* virus and treated with FA (n = 3). Data are shown as mean ± SD. ∗*P* < 0.05, ∗∗*P* < 0.01, ∗∗∗*P* < 0.001, ∗∗∗∗*P* < 0.0001 by one-way ANOVA (D-E), two-way ANOVA (L), and Student’s *t* test (B, G, J, Q and T). iWAT, inguinal white adipose tissue; gWAT, gonadal white adipose tissue; BAT, brown adipose tissue; BMP4, bone morphogenetic protein 4; TG, triglyceride; FA, fatty acid.

Given that fasting selectively promotes LCPUFA-enriched lipid storage in BAT, a central question is what upstream signals orchestrate this metabolic shift. In search of potential regulators, we focused on *Bmp4*, as a candidate factor whose mRNA and protein levels were uniquely upregulated in BAT (but not in WAT) specifically at the 20-h fasting time point (Fig. 2A, B). This induction was reversible, as re-feeding for 4 h returned BMP4 levels to baseline (Fig. 2C, E). This upregulation coincided temporally with the lipid droplet enlargement observed in BAT, suggesting a potential involvement of BMP4 in fasting-induced lipid metabolism.

To substantiate the function of BMP4 in BAT, we generated BAT-specific *Bmp4*-knockout mice (*Bmp4*^Δ*Ucp1*^) (Fig. 2F, G). When subjected to fasting, *Bmp4*^Δ*Ucp1*^ mice exhibited a marked reduction in BAT mass compared to *Bmp4*^fl/fl^ mice, despite no differences in body weight or other fat depot weights under basal conditions (Fig. 2H, J, Supplemental Fig. S2A, B). Critically, *Bmp4* depletion in BAT dramatically inhibited the fasting-induced lipid accumulation (Fig. 2K, L). Lipidomic analysis revealed that TGs were among the most depleted lipid classes, with nearly all detected TGs being lower in the knockout BAT (Fig. 2M, N, Supplemental Fig. S2C). Notably, the majority of these reduced TGs contained LCPUFA (Fig. 2N).

RNA sequencing of BAT from fasted mice showed that FA re-esterification genes were significantly downregulated upon *Bmp4* deletion (Fig. 2O, P). This reduction was confirmed by qPCR analysis (Fig. 2Q). Consistently, when exposed to FA, mature brown adipocytes with *Bmp4* knockdown showed reduced lipid droplet accumulation (Fig. 2R–T). Collectively, these results demonstrate that BMP4 directly facilitates FA re-esterification and storage, thereby driving lipid accumulation in BAT during fasting.

### BAT-specific *Bmp4* knockout improves whole-body energy expenditure through enhanced mitochondrial function

**Fig. 3:**
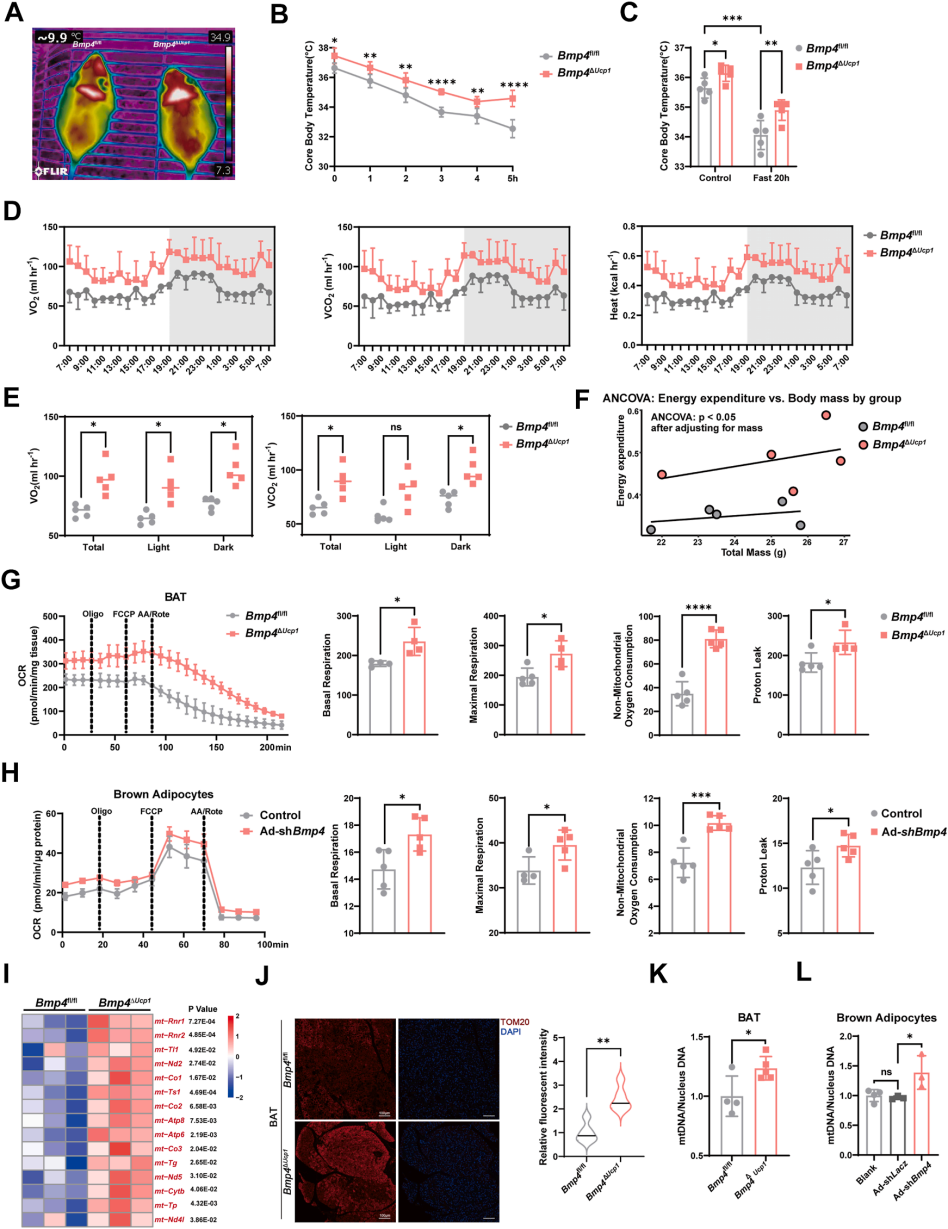
BAT-specific *Bmp4* knockout improves whole-body energy expenditure through enhanced mitochondrial function. A-B: Dorsal skin temperature and core body temperature of *Bmp4*^Δ*Ucp1*^ mice and *Bmp4*^fl/fl^ mice after 4°C cold exposure for 5 h without food supply (n = 5–7). C: Core body temperature of *Bmp4*^Δ*Ucp1*^ mice compared to *Bmp4*^fl/fl^ mice under fed or 20-h fasting conditions (n = 5). D-E: O_2_ consumption, CO_2_ production, and heat production analysis of *Bmp4*^Δ*Ucp1*^ mice and *Bmp4*^fl/fl^ mice at room temperature (n = 5). F: ANCOVA of energy expenditure with body weight as a covariate (n = 5). G: OCR of BAT from *Bmp4*^Δ*Ucp1*^ mice compared to *Bmp4*^fl/fl^ mice (n = 4–5). H: OCR of mature brown adipocytes infected with Ad-sh*LacZ* or Ad-sh*Bmp4* virus (n = 4–5). I: Heat map of mitochondria-related genes in BAT of *Bmp4*^Δ*Ucp1*^ mice versus *Bmp4*^fl/fl^ mice fasted for 20 h (n = 3). J: Representative IF images of TOM20 and quantification of relative fluorescent intensity in BAT from *Bmp4*^Δ*Ucp1*^ mice and *Bmp4*^fl/fl^ mice after a 20-h fast (n = 5). K: mtDNA copy number of BAT from *Bmp4*^Δ*Ucp1*^ mice and *Bmp4*^fl/fl^ mice (n = 4–5). L: mtDNA copy number of mature brown adipocytes infected with Ad-sh*LacZ* or Ad-sh*Bmp4* virus (n = 3–4). Data are shown as mean ± SD. ∗*P* < 0.05, ∗∗*P* < 0.01, ∗∗∗*P* < 0.001, ∗∗∗∗*P* < 0.0001 by one-way ANOVA (L), two-way ANOVA (B-C and E), ANCOVA (F), and Student’s *t* test (G-H, J-K). BAT, brown adipose tissue; ANCOVA, analysis of covariance; OCR, oxygen consumption rate; IF, immunofluorescence.

Next, we explored the functional consequences of impaired lipid accumulation in BAT. Infrared thermography revealed that *Bmp4*^Δ*Ucp1*^ mice maintained a higher dorsal surface temperature and core body temperature following cold exposure (Fig. 3A, B). Interestingly, *Bmp4*^Δ*Ucp1*^ mice exhibited a slightly higher basal body temperature, a difference that became even more pronounced after a 20-h fast (Fig. 3C), suggesting that the absence of *Bmp4* indeed plays a more significant role during the fasting period. Indirect calorimetry study showed that *Bmp4*^Δ*Ucp1*^ mice exhibited elevated systemic oxygen consumption (VO_2_), carbon dioxide release (VCO_2_), and heat production during both light and dark cycles when measured at room temperature (Fig. 3D, E). In accordance with the 2025 consensus guide on preclinical indirect calorimetry (40), we employed analysis of covariance (ANCOVA) to control for differences in body weight (Fig. 3F). These results confirmed that the knockout mice indeed exhibited significantly stronger energy metabolism. This elevated thermogenic output was underpinned by enhanced mitochondrial function. Our data show that *Bmp4* deficiency elevates basal and maximal mitochondrial respiration, and the elevated basal OCR appears to be driven primarily by enhanced uncoupled respiration rather than by greater ATP synthesis flux (Fig. 3G, H). Notably, *Bmp4* deficiency did not affect *Ucp1* expression levels in BAT, indicating that the enhancement of BAT thermogenic function does not occur via UCP1-dependent thermogenesis (Supplemental Fig. S3A, B). RNA-seq analysis revealed an upregulation of mitochondrial DNA-encoded genes in BAT of *Bmp4*^Δ*Ucp1*^ mice (Fig. 3I), suggesting enhanced mitochondrial biogenesis. This was confirmed by a rise in mitochondrial mass, evidenced by increased TOM20 immunofluorescence and a higher mitochondrial DNA copy number in knockout BAT (Fig. 3J–L). These results indicate an overall increase in thermogenesis in BAT from *Bmp4*^Δ*Ucp1*^ mice.

### *Bmp4* deficiency in BAT redirects FA metabolism from storage to oxidation

BAT exhibits remarkable metabolic flexibility, enabling it to switch its substrate utilization according to energy demand and nutritional status. During fasting, BAT rapidly shifts its energy substrate preference from glucose to FA (13). Experimental models in which lipid droplet accumulation in BAT is suppressed have been reported to exhibit increased glucose utilization by this tissue (19). To determine whether the impaired FA re-esterification in *Bmp4*^Δ*Ucp1*^ mice leads to a similar shift in fuel utilization, we examined systemic glucose metabolism. No differences were observed in serum glucose levels, BAT glucose transporter expression, 2-NBDG glucose uptake, whole-body glucose tolerance, insulin sensitivity or BAT glycogen content between *Bmp4*^Δ*Ucp1*^ mice and *Bmp4*^fl/fl^ mice (Supplemental Fig. S3C–I). These results indicate that the enhanced mitochondrial oxidative capacity in *Bmp4*^Δ*Ucp1*^ mice is not fueled by increased glucose utilization.

**Fig. 4:**
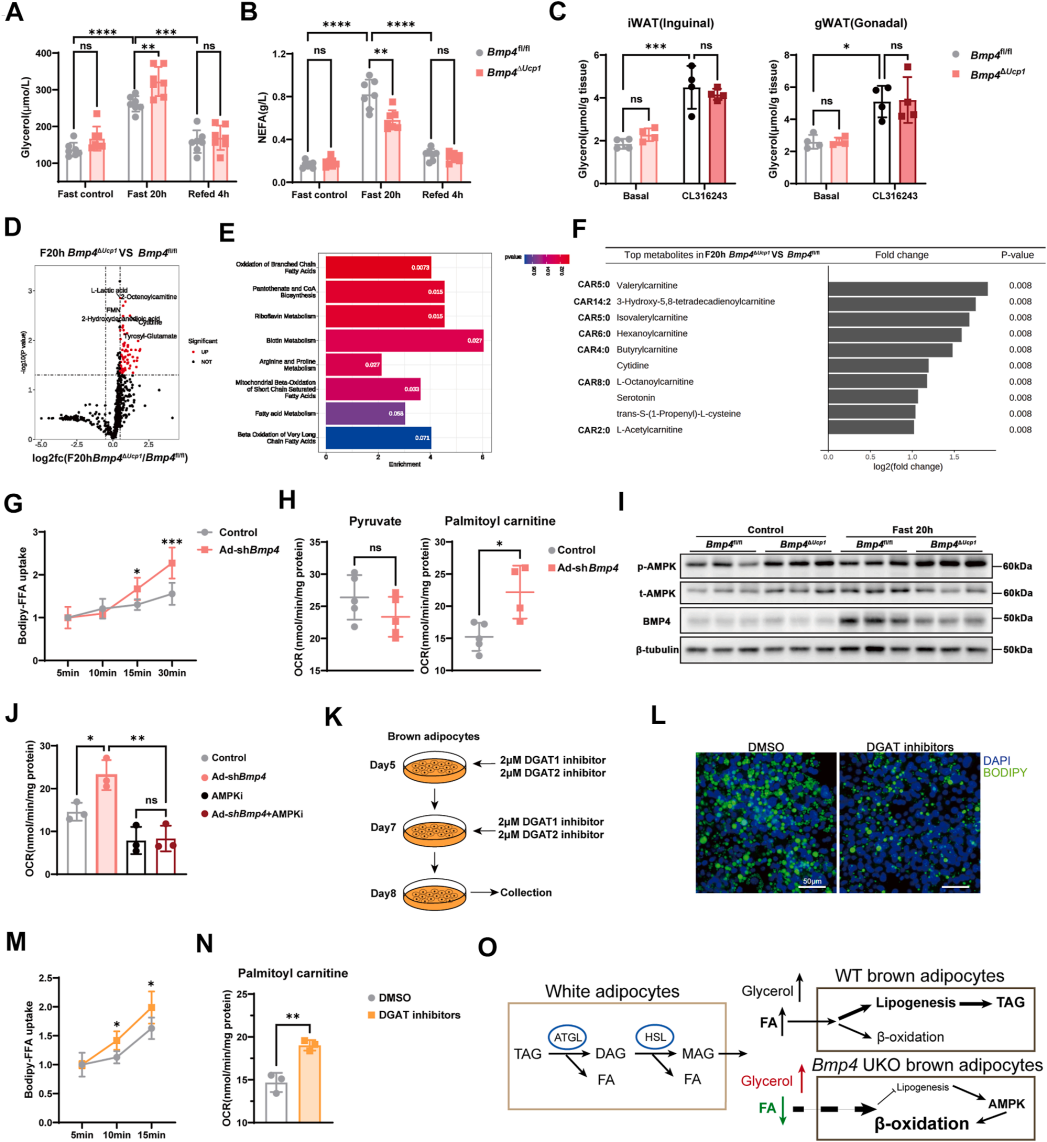
BAT-specific *Bmp4* knockout activates AMPK signaling and promotes FA utilization. A-B: Plasma levels of NEFA and glycerol in *Bmp4*^Δ*Ucp1*^ mice and *Bmp4*^fl/fl^ mice (n = 7). C: Glycerol release from iWAT and gWAT explants from *Bmp4*^Δ*Ucp1*^ mice and *Bmp4*^fl/fl^ mice under basal conditions and stimulation with the β_3_-adrenergic agonist CL316243 (n = 4). D: Volcano plot of metabolomics profiles in BAT from *Bmp4*^Δ*Ucp1*^ mice versus *Bmp4*^fl/fl^ mice after 20-h fasting (n = 5). E: Enrichment analysis of differentially abundant metabolite pathways from (D). F: Top metabolites in BAT from *Bmp4*^Δ*Ucp1*^ mice versus *Bmp4*^fl/fl^ mice after 20-h fasting (n = 5). G: FA uptake capacity of mature brown adipocytes infected with Ad-sh*LacZ* or Ad-sh*Bmp4* virus (n = 4). H: OCR of mature brown adipocytes infected with Ad-sh*LacZ* or Ad-sh*Bmp4* virus and assayed with pyruvate or palmityl carnitine as substrate (n = 4–5). I: p-AMPK and AMPK levels in BAT from *Bmp4*^Δ*Ucp1*^ mice and *Bmp4*^fl/fl^ mice under fed or 20-h fasting conditions (n = 3). J: OCR of mature brown adipocytes infected with Ad-sh*LacZ* or Ad-sh*Bmp4* virus and subsequent treatment with an AMPK inhibitor. Palmityl-carnitine was used as the oxidation substrate (n = 3). K: Schematic diagram of brown adipocyte treatment with DGAT1 and DGAT2 inhibitors. L: Representative BODIPY fluorescence staining in mature brown adipocytes treated with DMSO or DGAT inhibitors. M: FA uptake capacity in mature brown adipocytes treated with DMSO or DGAT inhibitors (n = 5–6). N: OCR of mature brown adipocytes treated with DMSO or DGAT inhibitors and assayed with palmityl carnitine as substrate (n = 3). O: Schematic diagram comparing FA flux in BAT between *Bmp4*^fl/fl^ mice (WT) and *Bmp4*^Δ*Ucp1*^ mice (*Bmp4* UKO) after fasting. Data are shown as mean ± SD. ∗*P* < 0.05, ∗∗*P* < 0.01, ∗∗∗*P* < 0.001, ∗∗∗∗*P* < 0.0001 by two-way ANOVA (A-C, G and M), one-way ANOVA (J) and Student’s *t* test (H and N). NEFA, non-esterified fatty acid; iWAT, inguinal white adipose tissue; gWAT, gonadal white adipose tissue; BAT, brown adipose tissue; OCR, oxygen consumption rate; FA, fatty acid.

Notably, serum glycerol levels were elevated in *Bmp4*^Δ*Ucp1*^ mice after 20 h of fasting (Fig. 4A), whereas serum FA levels were unexpectedly lower (Fig. 4B). To determine whether altered WAT lipolysis contributed to this discrepancy, we examined the expression of lipolysis-related genes in WAT (Supplemental Fig. S4A, B) and directly assessed WAT lipolytic capacity (Fig. 4C). No significant differences were observed, ruling out changes in WAT lipolysis as the cause. Given that glycerol and FA are consumed in equimolar amounts during TG synthesis via FA re-esterification (41), the combination of elevated glycerol and reduced FAs is consistent with impaired TG synthesis in BAT: reduced re-esterification leads to glycerol accumulation while unesterified FAs are cleared through alternative pathways such as oxidation, thereby lowering circulating FA levels. Indeed, untargeted metabolomics of BAT tissue showed a significant enrichment in pathways related to FA β-oxidation (Fig. 4D, E). Notably, metabolomic profiling revealed that six of the top ten most significantly upregulated metabolites were short-chain acylcarnitines (CAR) (Fig. 4F). Elevated short-chain CAR are considered as an indication of enhanced FA metabolism (42). These data suggest that upon *Bmp4* loss, FA uptake is increased but their esterification into TG is blocked, thereby diverting FAs toward mitochondrial oxidation.

Consistent with the in vivo findings, *Bmp4*-knockdown brown adipocytes showed increased FA uptake (Fig. 4G) and a selective increase in oxygen consumption only when palmitoyl-carnitine, not pyruvate, was provided as a substrate (Fig. 4H), confirming a specific enhancement of FA oxidative capacity.

### Impaired FA re-esterification activates AMPK signaling to promote FA catabolism

The coordinated increase in FA uptake and oxidation suggests the presence of a shared upstream regulatory mechanism. AMPK signaling has been widely reported to play a key role in suppressing lipid synthesis while promoting FA catabolism (43). We therefore examined AMPK activity in the *Bmp4*-deficient model. Western blot analysis confirmed elevated AMPK phosphorylation in BAT from *Bmp4*^Δ*Ucp1*^ mice (Fig. 4I). AMPK inhibition significantly attenuated the increase in cellular oxygen consumption rate induced by *Bmp4* silencing (Fig. 4J), indicating that AMPK signaling acts downstream of *Bmp4* loss to drive increased oxygen consumption. To establish that the metabolic shift was directly caused by defective FA re-esterification rather than other BMP4-dependent effects, we pharmacologically inhibited DGAT1/2 (Fig. 4K). This result was consistent with the effect observed in *Bmp4*-knockdown adipocytes: Reduced lipid droplet size, increased FA uptake and enhanced mitochondrial oxidation specifically with palmitoyl-carnitine (Fig. 4L–N). Moreover, AMPK activation did not alter the expression of re-esterification genes (Supplemental Fig. S4C). Therefore, AMPK signaling acts downstream of BMP4 and the re-esterification pathway it regulates.

Together, these data illustrate that *Bmp4* deficiency in BAT directly impairs FA re-esterification, leading to a substrate-specific metabolic remodeling characterized by enhanced FA uptake and oxidation, a process mediated at least in part by AMPK activation (Fig. 4O).

### AA acts as the effector lipid for AMPK activation following BMP4 deficiency

**Fig. 5:**
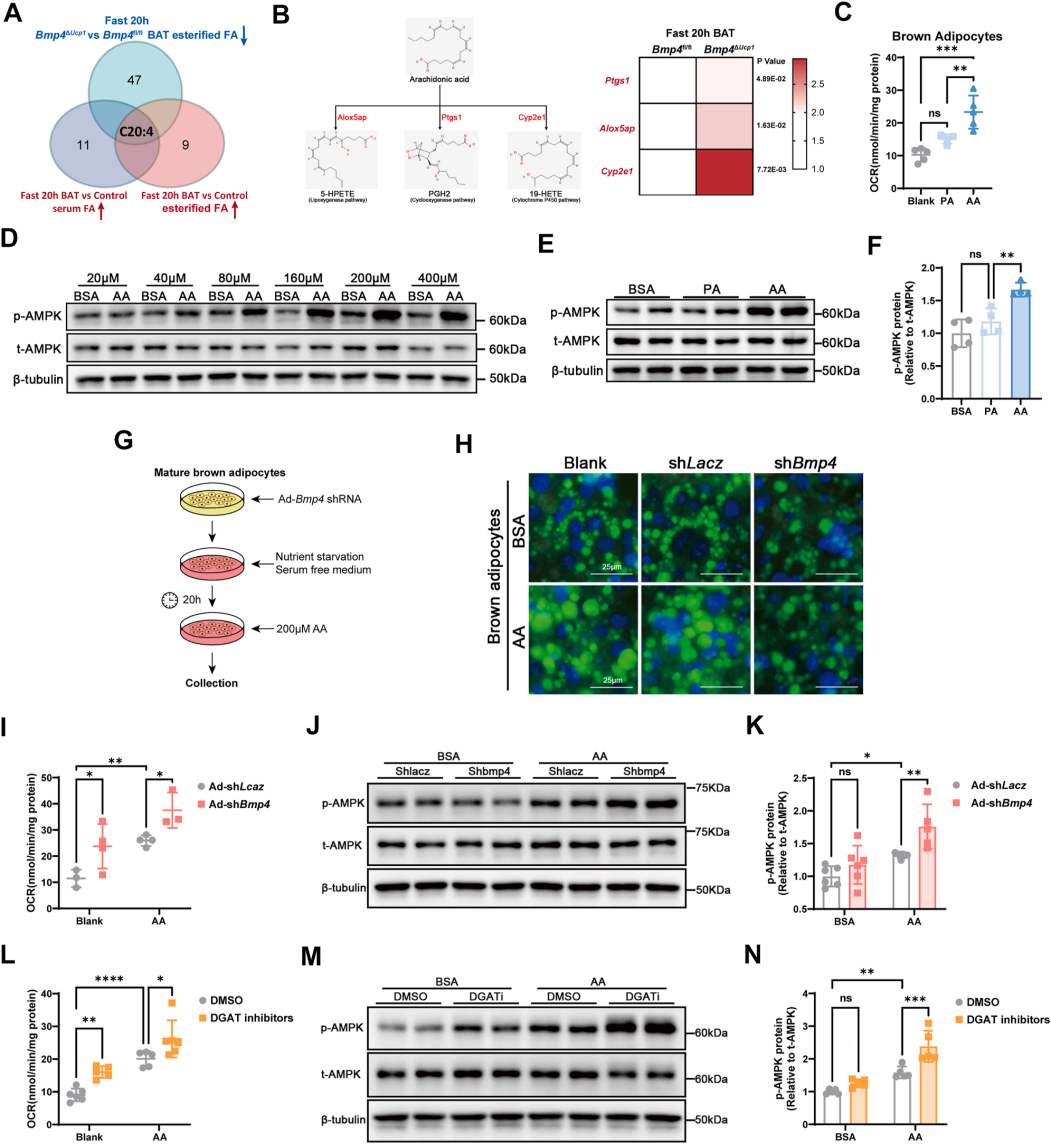
*Bmp4* deficiency stimulates mitochondrial function through the AA-AMPK axis. A: Schematic diagram of AA identification through a comparative analysis of multiple lipidomics datasets. B: Upregulated (red) genes relative to AA metabolism in RNA-sequencing of BAT from *Bmp4*^Δ*Ucp1*^ mice versus *Bmp4*^fl/fl^ mice after 20-h fasting. C: OCR of mature brown adipocytes assayed with 400 μM PA or AA as substrates (n = 4–5). D: p-AMPK level in mature brown adipocytes treated with a concentration gradient of AA. E-F: Western blot analysis and quantification of p-AMPK and AMPK levels in mature brown adipocytes treated with 200 μM PA or AA for 1 h (n = 4). G: Schematic of mature brown adipocytes infected with Ad-sh*LacZ* or Ad-sh*Bmp4* virus and exposed to high levels of AA. H: Representative BODIPY staining of mature brown adipocytes infected with Ad-sh*LacZ* or Ad-sh*Bmp4* and treated with BSA or AA. I: OCR of mature brown adipocytes assayed with 400 μM AA. The cells were infected with Ad-sh*LacZ* or Ad-sh*Bmp4* (n = 3–4). J-K: p-AMPK and AMPK levels in mature brown adipocytes treated with 200 μM AA for 1 h. The cells were infected with Ad-sh*LacZ* or Ad-sh*Bmp4* (n = 5–6). L: OCR of mature brown adipocytes assayed with 400 μM AA. The cells were pre-treated with DMSO or DGAT inhibitors (n = 5–6). M-N: p-AMPK and AMPK levels in mature brown adipocytes treated with 200 μM AA for 1 h. The cells were pre-treated with DMSO or DGAT inhibitors (n = 5). Data are shown as mean ± SD. ∗*P* < 0.05, ∗∗*P* < 0.01, ∗∗∗*P* < 0.001, ∗∗∗∗*P* < 0.0001 by one-way ANOVA (C and F) and two-way ANOVA (I, K, L, N). BAT, brown adipose tissue; AA, arachidonic acid; PA, palmitic acid; BMP4, bone morphogenetic protein 4; OCR, oxygen consumption rate.

To identify the upstream signal driving the metabolic shift from storage to oxidation, we focused on AMPK, a central energy sensor activated by low nutrient availability or specific metabolites (33, 44). During fasting, FAs mobilized from WAT are taken up by BAT and preferentially channeled into TG esterification prior to β-oxidation. When this FA esterification cycle is disrupted, unesterified FAs are redirected toward mitochondrial β-oxidation. Based on established evidence that FA binding to AMPK stimulates thermogenesis (33), we hypothesized that specific FAs species accumulate in the serum and BAT TG pool during fasting but are depleted in BAT under *Bmp4* knockout conditions. Candidate lipids were required to meet three stringent criteria: (i) elevation in serum upon fasting, (ii) enrichment in BAT TGs following fasting, and (iii) reduction in BAT TGs upon *Bmp4* loss. Arachidonic acid (AA) was the sole FA satisfying all three conditions (Fig. 5A), indicating its selective uptake and esterification in BAT during fasting are BMP4-dependent.

In addition, RNA sequencing revealed that the expression of enzymes initiating the three major AA metabolic pathways was increased in the BAT of *Bmp4*^Δ*Ucp1*^ mice (45) (Fig. 5B). Consistent with prior reports that AA is prone to being esterified to form TG under physiological conditions (46), our findings suggest that *Bmp4* deficiency directly inhibits FA esterification, leading to elevated free AA levels, which in turn indirectly activate downstream metabolic pathways.

When cells utilized AA instead of palmitic acid (PA) as a metabolic fuel, their OCR increased significantly (Fig. 5C). This suggests that inhibiting AA re-esterification itself may be sufficient to activate cellular energy metabolism. We found that in brown adipocytes, AA treatment induced a strong, concentration-dependent increase in AMPK phosphorylation within a short period (Fig. 5D–F, Supplemental Fig. S5A), whereas PA did not (Fig. 5E, F). The previous study has demonstrated that linoleic acid may allosterically activate AMPK similarly to AMP by directly binding the AMPK subunits in the liver (33). Molecular docking simulations suggested that AA also directly activates AMPK (Supplemental Fig. S5B). More concretely, AA binds the CBS3 site on AMPK, forming interactions similar to those of AMP, with a predicted binding energy of −5.18 kcal/mol, indicating potential allosteric activation. This binding mode overlaps with that of AMP (47, 48) (Supplemental Fig. S5C), suggesting AA may allosterically activate AMPK by stabilizing the active conformation and protecting Thr172 from dephosphorylation.

### Liberating AA activates AMPK signaling and enhances mitochondrial function

Having established that AA activates AMPK and serves as an efficient metabolic substrate in BAT, we next asked how its physiological function is altered when re-esterification is impaired. Specifically, we sought to determine whether changes in AA handling under this condition could explain the enhanced energy metabolism observed in *Bmp4*-deficient mice. To address this, we treated *Bmp4*-knockdown brown adipocytes with exogenous AA (Fig. 5G). The AA-induced enlargement of lipid droplets observed in controls was blunted upon *Bmp4* loss (Fig. 5H). The OCR was higher in *Bmp4*-knockdown brown adipocytes when AA was supplied as the substrate (Fig. 5I). Most importantly, AA further increased p-AMPK levels in *Bmp4*-knockdown cells (Fig. 5J, K). Accordingly, pharmacological inhibition of FA re-esterification with DGAT inhibitors in brown adipocytes resulted in a similar phenotype (Fig. 5L–N).

In summary, our results position AA as a key metabolic mediator in brown adipocytes. With FA re-esterification inhibited via *Bmp4* deletion, AA potently enhances thermogenic capacity in brown adipocytes through direct allosteric activation of AMPK.

### *Bmp4* overexpression promotes lipid accumulation in BAT and opposes knockout-associated phenotypes

**Fig. 6:**
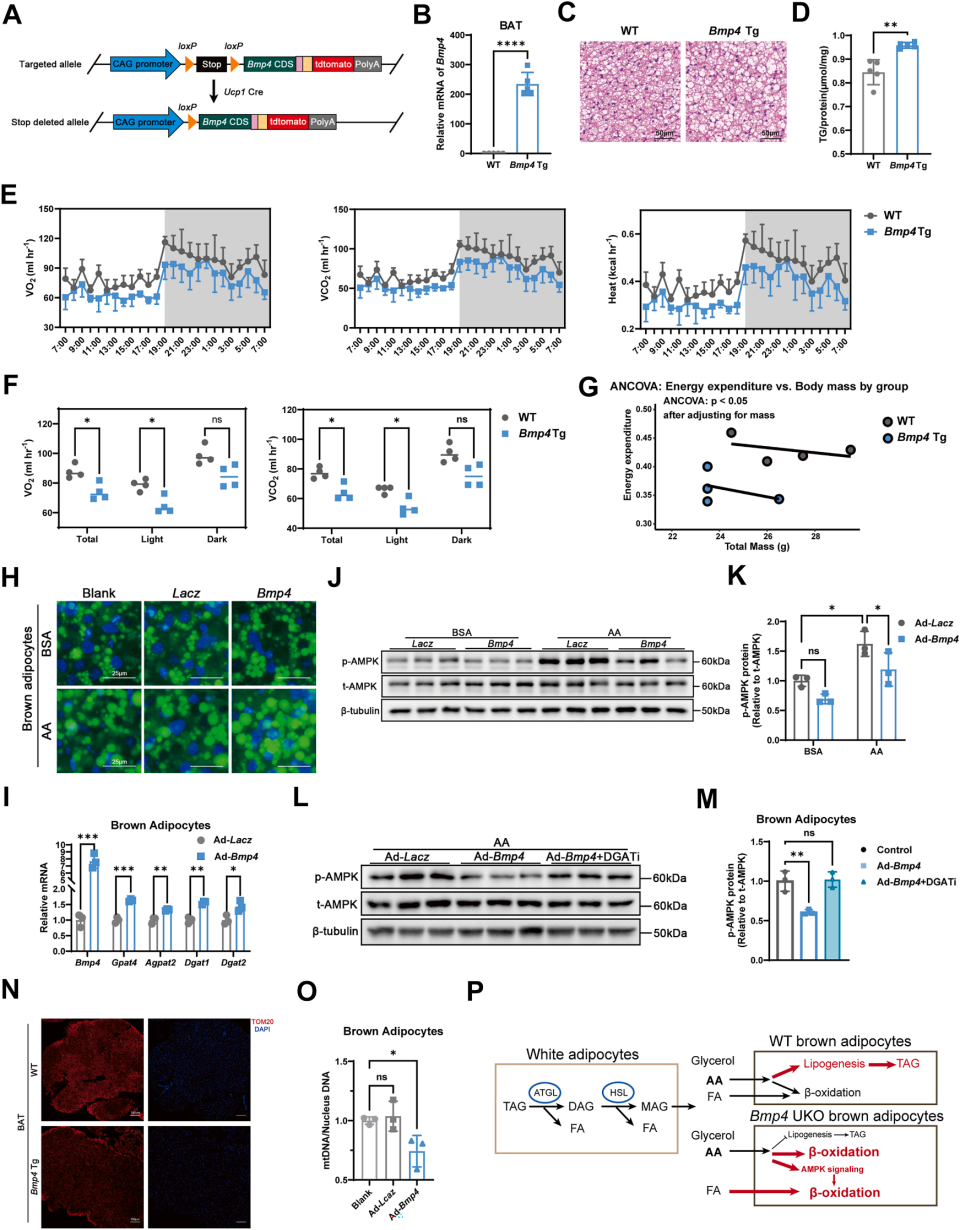
*Bmp4* overexpression promotes lipid accumulation in BAT and opposes knockout-associated phenotypes. A: Schematic of the BAT-specific *Bmp4* transgene (*Bmp4* Tg) mouse model. B: Relative mRNA levels of *Bmp4* in BAT (n = 5). C: Representative H&E-staining microscopic images of BAT from mice under fed conditions. (D) Quantitative analyses of TG content in BAT (n = 5). E-F: O_2_ consumption, CO_2_ production, and heat production analysis of *Bmp4* Tg mice and wild-type (WT) mice at room temperature (n = 4). G: Analysis of covariance (ANCOVA) of energy expenditure with body weight as a covariate (n = 4). H: Representative BODIPY staining of mature brown adipocytes infected with Ad-*LacZ* or Ad-*Bmp4* and treated with BSA or AA. I: Relative mRNA levels of *Bmp4* and FA esterification genes in mature brown adipocytes (n = 3). J-K: p-AMPK and AMPK levels in mature brown adipocytes treated with 200 μM AA for 1 h. The cells were infected with Ad-*LacZ* or Ad-*Bmp4* (n = 3). L-M: p-AMPK and AMPK levels were measured in mature brown adipocytes infected with Ad-*LacZ* or Ad-*Bmp4* (with or without treatment with DGAT inhibitors) and then stimulated with 200 μM AA for 1 h (n = 3). N: Representative IF images of TOM20 in BAT from *Bmp4* Tg mice and wild-type (WT) mice. O: mtDNA copy number of mature brown adipocytes infected with Ad-*LacZ* or Ad-*Bmp4* virus (n = 3). P: Schematic diagram comparing AA flux in BAT between wild-type (WT) and *Bmp4*^Δ*Ucp1*^ mice (*Bmp4* UKO) after fasting. Data are shown as mean ± SD. ∗*P* < 0.05, ∗∗*P* < 0.01, ∗∗∗*P* < 0.001, ∗∗∗∗*P* < 0.0001 by one-way ANOVA (M and O), two-way ANOVA (F, K), ANCOVA (G) and Student’s *t* test (B, D and I). BAT, brown adipose tissue; TG, triglyceride; BMP4, bone morphogenetic protein 4; AA, arachidonic acid; IF, immunofluorescence.

Finally, we generated a BAT-specific *Bmp4* overexpression mouse model to validate that BMP4 indeed promotes lipid accumulation in BAT by modulating the re-esterification process and consequently affects BAT thermogenic function (Fig. 6A, B). At the tissue level, BAT from *Bmp4* Tg mice displayed pronounced lipid droplet accumulation (Fig. 6C, D). Consistent with a function opposite to that seen in knockout mice, *Bmp4* transgenic (*Bmp4* Tg) mice exhibited a systemic reduction in energy expenditure; indirect calorimetry revealed that *Bmp4* Tg mice exhibited consistently reduced whole-body VO_2_, VCO_2_ and heat production (Fig. 6E–G) throughout both the light and dark cycles. This indicates that *Bmp4* overexpression reduces whole-body energy metabolism, opposing the phenotype observed in *Bmp4*^Δ*Ucp1*^ mice.

Consistent with the findings in *Bmp4*-knockdown brown adipocytes, treatment of *Bmp4*-overexpressing brown adipocytes with AA revealed larger lipid droplets (Fig. 6H) and increased mRNA levels of re-esterification genes (Fig. 6I). Moreover, *Bmp4*-overexpressing brown adipocytes exhibited suppression of the AMPK signaling pathway, both before and after AA treatment (Fig. 6J, K). DGAT inhibitor treatment rescued the AMPK signaling deficit induced by *Bmp4* overexpression to control levels, demonstrating that BMP4 regulates AMPK pathway activation via modulation of AA re-esterification (Fig. 6L, M).

Corresponding to the knockout phenotype, *Bmp4* Tg mice showed reduced mitochondrial content in BAT as evidenced by immunofluorescence (Fig. 6N). Consistently, *Bmp4* overexpression in mature brown adipocytes led to decreased mtDNA levels, confirming a reduction in mitochondrial number (Fig. 6O).

Taken together, these results demonstrate that BMP4 acts within brown adipocytes to regulate the re-esterification signaling pathway. By modulating the extent of AA re-esterification, *Bmp4* deficiency influences the activation of the intracellular AMPK pathway, thereby regulating cellular FAs flux and energy metabolism (Fig. 6P).

## Discussion

Our study elucidates a previously unrecognized signaling axis that governs the fate of FA in BAT during fasting. We identify BMP4 as a fasting-induced regulator that promotes lipid storage by driving the re-esterification of specific FAs, notably AA. When this pathway is disrupted, liberated AA activates AMPK, redirecting metabolic flux toward oxidation. This BMP4-AA-AMPK axis establishes re-esterification not merely as a storage endpoint, but as a critical regulatory node that dynamically allocates lipid substrates between storage and combustion in BAT.

This concept of regulated storage helps reconcile long-standing controversies regarding the role of lipid droplets in BAT thermogenesis. Studies under conditions like cold exposure have yielded seemingly conflicting results, with some suggesting lipid storage in BAT is dispensable and others indicating it is essential (19, 20, 21, 23, 49, 50). Our findings in the fasting context introduce a unifying perspective: Lipid droplets act as a tunable brake on thermogenic capacity. Under fasting, their expansion is part of an adaptive, energy-conserving program, dampening excessive heat production. Inhibiting their formation, as in our *Bmp4*-knockout or DGAT-inhibition models, releases this brake, leading to a hypermetabolic state. While our study defines the acute metabolic effects of inhibiting BAT re-esterification during fasting, the long-term physiological impact of this manipulation remains an open question. It would be intriguing for future studies to investigate how blocking this specific lipid storage pathway affects whole-body metabolism over repeated cycles of intermittent fasting. Such research will be crucial to fully elucidate the physiological significance of the selective lipid sequestration in BAT during nutrient deprivation, and to determine whether it represents a viable therapeutic target for modulating energy balance.

Several studies have reported that inhibiting lipid accumulation stimulates catabolic pathways in brown adipocytes. The endoplasmic reticulum-localized protein calsyntenin-3 beta (CLSTN3β) maintains brown adipocyte thermogenic activity by limiting lipid droplet expansion (51), and DGAT inhibitor treatment activates AMPK signaling pathway and enhances mitochondrial oxidative respiration (52), yet the trigger for AMPK activation remained unknown. Our study identifies AA as a critical metabolite regulator of energy metabolism in brown adipocytes. Previous studies have demonstrated the pro-inflammatory effects of AA (53), and some AA metabolites have also been reported to enhance BAT thermogenesis (54, 55). Here, we show that inhibiting AA re-esterification activates AMPK signaling and enhances mitochondrial oxidative capacity in fasting BAT. This further demonstrates the important role of AA in regulating BAT energy metabolism. Thus, AA acts as a metabolic switch: its compartmentalization into lipid droplets maintains anabolic balance, while its release redirects metabolism toward oxidation.

Our data illuminate a fundamental dichotomy in the physiological purpose of lipid droplets between WAT and BAT. In WAT, re-esterification serves as a long-term energy reservoir and a buffer against systemic lipotoxicity (46). In contrast, lipid droplets in BAT are dynamic and facultative. Cold exposure enhances catabolic activity, leading to reduced lipid droplets (23, 56). In contrast, during fasting, brown adipocytes initially shift toward lipid droplet expansion. This uptake is likely an active process rather than a consequence of passive accumulation. During early fasting, lipolysis in WAT releases a mixture of FAs into circulation (57). At this moment, BAT selectively take up LCPUFA, such as AA, which are efficient substrates for oxidation. We hypothesize that the physiological rationale is to prepare for prolonged nutrient scarcity: as fasting extends and systemic lipid reserves from WAT decline, BAT can then mobilize these pre-stored, high-yield substrates. This arrangement ensures that even during late-stage fasting, the thermogenic tissue retains access to efficient fuels to sustain its critical heat production and metabolic regulatory functions, thereby providing a final safeguard for systemic energy homeostasis.

It has been widely reported that overexpression of *Bmp4* in BAT and brown adipocytes impairs the metabolic functions (26, 29). For instance, systemic AAV-mediated *Bmp4* overexpression was shown to increase BAT lipid content, a phenotype previously attributed to impaired lipolysis. While this observation aligns with our finding of reduced lipid accumulation in *Bmp4*^Δ*Ucp1*^ mice, our data pinpoint a different underlying mechanism. Our findings establish that BMP4 promotes lipid accumulation in BAT primarily through enhancing FA esterification. This interpretation is further supported by the fact that β3-agonist treatment activated lipolysis and reduced lipid droplet size both in wild-type and BMP4-treated mice, suggesting that impaired lipolysis is not the main driver of BMP4-induced lipid accumulation (27). Our premise for investigating BMP4 under fasting conditions stemmed from our prior observation that it upregulates SCD1, a key lipogenic enzyme, in adipocytes, pointing to a potential role in regulating lipid anabolism (28). This prompted us to employ the fasting model, which robustly induces lipid accumulation in BAT, as an ideal system to test whether BMP4 governs the expression of lipogenic or re-esterification genes. Our results indicate that BMP4 likely directly upregulates genes involved in FA re-esterification, thereby diverting FAs away from mitochondrial β-oxidation. This is consistent with our observation that the expression of key β-oxidation genes, *Cpt1b* and *Cpt2*, was unaltered in BAT of *Bmp4* knockout mice (data not shown), excluding a direct effect on the core β-oxidation machinery. Additionally, we noted that *Bmp4* deficiency led to the upregulation of genes involved in AA metabolism. We currently interpret this as an indirect regulatory effect. Specifically, the loss of *Bmp4* inhibits AA re-esterification, resulting in an accumulation of intracellular AA, which in turn activates AA metabolic pathways. However, this transcriptional response occurs downstream of AMPK activation, as we observed robust AMPK signaling within one hour of AA treatment in *Bmp4*-deficient cells, suggesting that AMPK activation precedes the metabolic gene reprogramming. Therefore, our study reveals a previously underappreciated anabolic mechanism of BMP4 in BAT, demonstrating its essential role in driving FA re-esterification to modulate the tissue’s thermogenic capacity.

In conclusion, our work identifies the BMP4-AA-AMPK axis as a critical mechanism that directs FAs toward storage or oxidation in BAT during fasting. Our findings extend beyond revealing a novel signaling role for AA; they redefine re-esterification in BAT as an active regulatory checkpoint, not a passive metabolic branch. This checkpoint determines the fate of lipid substrates. Consequently, BAT emerges not merely as a thermogenic organ, but as a dynamic regulator of energy flow. Based on this new understanding, our findings suggest a potential practical application. Intermittent fasting improves metabolism partly by promoting WAT browning (1, 4, 5). Our results propose an additional mechanism and a synergistic strategy: targeted inhibition of BAT re-esterification during fasting windows could further enhance mitochondrial function, thereby amplifying the benefits of fasting on whole-body energy expenditure. Additionally, activating BAT thermogenesis to enhance whole-body metabolism has emerged as a promising strategy for treating obesity and associated metabolic disorders. Our study suggests that inhibiting BAT re-esterification to boost its oxidative metabolism may offer a new therapeutic approach for these conditions.

## Data availability

Further information and requests for resources and reagents should be directed to and will be fulfilled by the lead contact, Dr Yan Tang (yantang@fudan.edu.cn). Bulk RNA-seq data are available in the NCBI Gene Expression Omnibus (GEO) under accession number GSE308935.

## Supplemental data

This article contains supplemental data.

## Conflict of interest

The authors declare that they have no conflicts of interest with the contents of this article.
