# Supplementary material for: Deleting Bmp4 in brown fat unlocks an arachidonic acid-AMPK axis to boost energy expenditure
Source: J Lipid Res. 2026 Jul 15;67(8):101103. doi: 10.1016/j.jlr.2026.101103 (PMC13487297; doi:10.1016/j.jlr.2026.101103)
Supplement: Supplementary file 1 [file mmc1.docx]

SUPPLEMENTAL INFORMATION

**Deleting *Bmp4* in brown fat unlocks an arachidonic acid-AMPK axis**

**to boost energy expenditure**

Yan-Jue Song^1^, Ting Meng^1^, Zhen-Yu Xu^1^, Ting Zhang^2^, Zhao-Ning Wang^1^, Dai-Chen Yao^1^, Li-Jie Yang^1^, Ping Wu^1^, Shu-Wen Qian^1^, Yang Liu^1^, **Qi-Qun Tang**^1^✉, **Yan Tang**^1,3^✉

^1^Key Laboratory of Metabolism and Molecular Medicine, Ministry of Education, Department of Biochemistry and Molecular Biology of School of Basic Medical Sciences and Department of Endocrinology and Metabolism of Zhongshan Hospital, Fudan University, Shanghai 200032, China

^2^Department of Laboratory Medicine, Shanghai Fourth Rehabilitation Hospital, Shanghai 200032, China

^3^Lead contact

✉Corresponding authors

[yantang@fudan.edu.cn](mailto:yantang@fudan.edu.cn). Room 4-030, 2# Research Building, 138 Yixueyuan Road, Shanghai 200032, P. R. China. 86-21-54237198

[qqtang@shmu.edu.cn](mailto:qqtang@shmu.edu.cn). Room 4-008, 2# Research Building, 138 Yixueyuan Road, Shanghai 200032, P. R. China. 86-21-54237198

**Supplemental Figure S1. Physiological parameters of adipose tissues and liver under fasting or cold exposure**

(**A-B**) p-HSL and HSL level in iWAT and gWAT from mice fasted for 0, 12, 24 and 48 hours (n=3). (**C-D**) Representative H&E-staining microscopic images of BAT and liver from mice fasted for 0, 12, 24 and 48 hours. (**E**) Statistical analysis of lipid droplet diameters in BAT (n=5). (**F**) Relative mRNA levels of *Ucp1* in BAT from mice fasted for 0, 12, 16, 20 and 24 hours (n=5). (**G**) Representative H&E-staining microscopic images of BAT from mice under cold exposure for 0, 6, 12, 16, 20, and 24 hours. (**H**) Relative mRNA levels of *Ucp1*, *Pgc1α*, *Acox1* and *Cpt1b* in BAT (n=4). (**I**) Relative mRNA levels of *Gpat4*, *Dgat1* and *Dgat2* in three types of adipose tissues and the liver isolated from mice after 20 hours of cold exposure compared with 0-hour controls (n=4). Data are shown as mean ± SD. ∗p < 0.05, ∗∗p < 0.01, ∗∗∗p < 0.001, ∗∗∗∗p < 0.0001 by one-way ANOVA analysis (**B**, **E, F** and **H**) and Student’s t-test (**I**).

iWAT, inguinal white adipose tissue; gWAT, gonadal white adipose tissue; BAT, brown adipose tissue.

**Supplemental Figure S2. Body weight and mass of three adipose tissues in *Bmp4*^Δ^*^Ucp1^* mice were comparable to *Bmp4*^fl/fl^** **mice under basal conditions. Comparative lipidomic analysis of BAT from *Bmp4*^Δ^*^Ucp1^* mice versus *Bmp4*^fl/fl^** **mice.**

(**A**) Representative images of adipose tissues from *Bmp4*^fl/fl^ and *Bmp4*^Δ^*^Ucp1^* mice under fed conditions. (**B**) Body weight and weights of fat pads of *Bmp4*^fl/fl^ and *Bmp4*^Δ^*^Ucp1^* mice under fed conditions (n=6-7). (**C**) Top 10 lipids with the highest up- and down-regulated fold changes from the lipidomics analysis in Figure 2**M**-**N**. Data are shown as mean ± SD. ns p > 0.05 by Student’s t-test.

**Supplemental Figure S3.** ***Bmp4* deficiency does not affect glucose metabolism in BAT.**

(**A**) Relative mRNA levels of *Ucp1* in BAT from *Bmp4*^Δ^*^Ucp1^* mice and *Bmp4*^fl/fl^ mice (n=5). (**B**) UCP1 level in BAT from *Bmp4*^Δ^*^Ucp1^* mice and *Bmp4*^fl/fl^ mice (n=3). (**C**) Plasma glucose levels in *Bmp4*^Δ^*^Ucp1^* mice and *Bmp4*^fl/fl^ mice (n=7). (**D**) Relative mRNA levels of *Glut1* and *Glut4* in BAT from *Bmp4*^Δ^*^Ucp1^* mice and *Bmp4*^fl/fl^ mice (n=4-6). (**E**) 2-NBDG glucose uptake of mature brown adipocytes infected with Ad-sh*LacZ* or Ad-sh*Bmp4* virus (n=3). (**F**) Plasma glucose levels in *Bmp4*^Δ^*^Ucp1^* mice and *Bmp4*^fl/fl^ mice during glucose tolerance test (GTT) and the area under curve analysis (n=5-9). (**G**) Plasma glucose levels in *Bmp4*^Δ^*^Ucp1^* mice and *Bmp4*^fl/fl^ mice during insulin tolerance test (ITT) and the area under curve analysis (n=5-9). (**H**) Representative PAS-staining microscopic images of BAT from *Bmp4*^Δ^*^Ucp1^* mice and *Bmp4*^fl/fl^ mice. (**I**) GYS1 and BMP4 level in BAT from *Bmp4*^Δ^*^Ucp1^* mice and *Bmp4*^fl/fl^ mice after 20-hour fasting and 4-hour refeeding (n=3). Data are shown as mean ± SD. ∗p < 0.05, ∗∗p < 0.01, ∗∗∗p < 0.001, ∗∗∗∗p < 0.0001 by Student’s t-test (**A** and analysis of area under curve in **F**, **G**) and two-way ANOVA analysis (**C**-**E**).

**Supplemental Figure S4. *Bmp4* knockout maintains WAT lipolysis unchanged.**

(**A**) Relative mRNA levels of *Atgl* and *Hsl* in iWAT and gWAT from *Bmp4*^Δ^*^Ucp1^* mice and *Bmp4*^fl/fl^ mice (n=5-7). (**B**) p-HSL and HSL levels in iWAT and gWAT from *Bmp4*^Δ^*^Ucp1^* mice and *Bmp4*^fl/fl^ mice (n=4). (**C**) Relative mRNA levels of *Gpat4, Agpat2, Dgat1* and *Dgat2* in brown adipocytes treated with AMPK inhibitor after infection with Ad-sh*LacZ* or Ad-sh*Bmp4* (n=3). Data are shown as mean ± SD. ∗p < 0.05, ∗∗p < 0.01, ∗∗∗p < 0.001, ∗∗∗∗p < 0.0001 by Student’s t-test (**A** and **B**) and one-way ANOVA analysis (**C**).

iWAT, inguinal adipose tissue; gWAT, gonadal adipose tissue.

**Supplemental Figure S5. AA directly binds to AMPK and promotes its phosphorylation.**

(**A**) p-AMPK and AMPK levels in mature brown adipocytes treated with 200 μM AA for 0, 1, 2 and 4 hours (n=4). (**B**-**C**) Molecular docking analysis of AA and AMPK structure. Data are shown as mean ± SD. ∗p < 0.05, ∗∗p < 0.01, ∗∗∗p < 0.001, ∗∗∗∗p < 0.0001 by one-way ANOVA analysis.

**Table S1:**

**Primer sequences used for quantitative real-time PCR (qPCR)**

| **Genes** | **Primer sequence-Forward** | **Primer sequence-Reverse** |
| --- | --- | --- |
| *Acc* | TGTACAAGCAGTGTGGGCTGGCT | CCACATGGCCTGGCTTGGAGGG |
| *18s* | CGCCGCTAGAGGTGAAATTCT | CATTCTTGGCAAATGCTTTCG |
| *Fatp1* | CCGTATCCTCACGCATGTGT | CTCCATCGTGTCCTCAATGAC |
| *Dgat1* | GGAATATCCCCGTGCACAA | CATTTGCTGCTGCCATGTC |
| *Dgat2* | CCGCAAAGGCTTTGTGAA | GGAATAAGTGGGAACCAGATCAG |
| *Gpat4* | AGCTTGATTGTCAACCTCCTG | CCGTTGGTGTAGGGCTTGT |
| *Acox1* | TAACTTCCTCACTCGAAGCCA | AGTTCCATGACCCATCTCTGTC |
| *Cpt1β* | GCACACCAGGCAGTAGCTTT | CAGGAGTTGATTCCAGACAGGTA |
| *Bmp4* | TTCCTGGTAACCGAATGCTGA | CCTGAATCTCGGCGACTTTTT |
| *Cebpα* | CAAGAACAGCAACGAGTACCG | GTCACTCGTCAACTCCAGCAC |
| *Cytob* | CCTTCATGTCGGACGAGGCTT | TGCTGTGGCTATGACTGCGAA |
| *Cd36* | ATGGGCTGTGATCGGAACTG | GTCTTCCCAATAAGCATGTCTCC |
| *Ucp1* | ATCAGGGTATCCTCTCCCCAG | CTGAGTGAGGCAAAGCTGATTT |
| *Pgc1α* | TATGGAGTGACATAGAGTGTGCT | CCACTTCAATCCACCCAGAAAG |
| *Agpat2* | CAGCCAGGTTCTACGCCAAG | TGATGCTCATGTTATCCACGGT |
| *Glut1* | GGCCAGATTCATCAACTGGAT | GCTCCAAGGCTGTACCCTAAG |
| *Glut4* | GTGACTGGAACACTGGTCCTA | CCAGCCAGTTGCATTGTAG |
| *Gpam* | ACAGTTGGCACAATAGACGTTT | CCTTCCATTTCAGTGTTGCAGA |
| *Agpat1* | TAAGATGGCCTTCTACAACGGC | CCATACAGGTATTTGACGTGGAG |
